# Risk of cardiovascular events after an exacerbation of chronic obstructive pulmonary disease: results from the EXACOS-CV cohort study using the PHARMO Data Network in the Netherlands

**DOI:** 10.1186/s12931-023-02601-4

**Published:** 2023-11-21

**Authors:** Karin M. A. Swart, Brenda N. Baak, Louise Lemmens, Fernie J. A. Penning-van Beest, Camilla Bengtsson, Muriel Lobier, Fabian Hoti, Dina Vojinovic, Lindy van Burk, Kirsty Rhodes, Edeltraut Garbe, Ron M. C. Herings, Clementine Nordon, Sami O. Simons

**Affiliations:** 1 PHARMO Institute for Drug Outcomes Research, Utrecht, The Netherlands; 2 IQVIA, Real World Solutions, Solna, Sweden; 3 IQVIA, Real World Solutions, Espoo, Finland; 4 IQVIA, Real World Solutions, Amsterdam, The Netherlands; 5 AstraZeneca, The Hague, The Netherlands; 6 AstraZeneca, Cambridge, UK; 7 Leibniz Institute for Prevention Research and Epidemiology – BIPS, Bremen, Germany; 8 Amsterdam Public Health Research Institute, Amsterdam UMC Location Vrije University Amsterdam, Amsterdam, The Netherlands; 9 Department of Respiratory Medicine, NUTRIM School of Nutrition and Translational Research in Metabolism, Maastricht University Medical Center, Maastricht, The Netherlands

**Keywords:** COPD, Chronic obstructive pulmonary disease, Cardiopulmonary risk, Exacerbation, Retrospective cohort study

## Abstract

**Background:**

People living with chronic obstructive pulmonary disease (COPD) have an increased risk of experiencing cardiovascular (CV) events, particularly after an exacerbation. Such CV burden is not yet known for incident COPD patients. We examined the risk of severe CV events in incident COPD patients in periods following either moderate and/or severe exacerbations.

**Methods:**

Persons aged ≥ 40 years with an incident COPD diagnosis from the PHARMO Data Network were included. Exposed time periods included 1–7, 8–14, 15–30, 31–180 and 181–365 days following an exacerbation. Moderate exacerbations were defined as those managed in outpatient settings; severe exacerbations as those requiring hospitalisation. The outcome was a composite of time to first severe CV event (acute coronary syndrome, heart failure decompensation, cerebral ischaemia, or arrhythmia) or death. Hazard ratios (HR) were estimated for association between each exposed period and outcome.

**Results:**

8020 patients with newly diagnosed COPD were identified. 2234 patients (28%) had ≥ 1 exacerbation, 631 patients (8%) had a non-fatal CV event, and 461 patients (5%) died during a median follow-up of 36 months. The risk of experiencing the composite outcome was increased following a moderate/severe exacerbation as compared to time periods of stable disease [range of HR: from 15.3 (95% confidence interval 11.8–20.0) in days 1–7 to 1.3 (1.0–1.8) in days 181–365]. After a moderate exacerbation, the risk was increased over the first 180 days [HR 2.5 (1.3–4.8) in days 1–7 to 1.6 (1.3–2.1) in days 31–180]. After a severe exacerbation, the risk increased substantially and remained higher over the year following the exacerbation [HR 48.6 (36.9–64.0) in days 1–7 down to 1.6 (1.0–2.6) in days 181–365]. Increase in risk concerned all categories of severe CV events.

**Conclusions:**

Among incident COPD patients, we observed a substantial risk increase of severe CV events or all-cause death following either a moderate or severe exacerbation of COPD. Increase in risk was highest in the initial period following an exacerbation. These findings highlight the significant cardiopulmonary burden among people living with COPD even with a new diagnosis.

**Supplementary Information:**

The online version contains supplementary material available at 10.1186/s12931-023-02601-4.

## Introduction

Chronic obstructive pulmonary disease (COPD) is a heterogeneous respiratory condition, characterised by chronic respiratory symptoms due to abnormalities of the airways and/or alveoli that cause persistent, often progressive airflow obstruction [1]. Exacerbations of COPD negatively impact prognosis, often leading to more frequent and severe exacerbations, accelerated loss of lung function, and amplifying risk of mortality [2].

Persons with COPD have a 2 to threefold increased risk of cardiovascular (CV) disease compared to persons without COPD, regardless of age, sex, smoking status or other confounders [3]. In particular, time periods following an exacerbation have been identified as periods wherein patients are at an increased risk of incident severe CV events [4]. Although many studies have examined the cardiovascular risk in COPD patients, to our knowledge, these have focused on prevalent, severe COPD patients, i.e. either from randomized controlled trials or selected because they had a CV event. The association between exacerbations and cardiovascular risk has not been studied in newly diagnosed patients. Importantly, newly diagnosed patients may already be experiencing exacerbations [5] and this may confer an increased risk of cardiovascular disease already early after diagnosis.

The aims of this study were to determine in a population of patients newly diagnosed with COPD, (1) the association between a moderate or severe exacerbation and a composite of non-fatal severe CV events or all-cause death; (2) the association between a moderate or severe exacerbation and the individual types of CV events of interest separately; (3) the association with the composite outcome for moderate and severe exacerbations separately; and (4) the association with the outcome with the exacerbation recurrence, for each of the subsequent first, second, and third exacerbation.

## Methods

This study is part of the EXAcerbations of COPD and their OutcomS on CardioVascular disease (EXACOS-CV) programme. The overall study design and analytical approaches were detailed previously [6].

### Study design and population

This retrospective cohort study used data from the PHARMO Data Network from the Netherlands [7]. The source population included people who were registered as patients with participating GP’s (in total 20% of the Dutch population) and whose GP records could be linked to hospital and out-patient pharmacy records, irrespective of whether they were hospitalized or received medication between 1 January 2014 and 31 December 2018 (45% of the GP population). Individuals aged ≥ 40 years, with an incident, GP-reported diagnosis of COPD (International Classification of Primary Care (ICPC) R95 or R91.01) in this period were selected. The entire available lookback period was used to confirm the incident nature of COPD diagnosis. The date of COPD diagnosis was cohort entry date (CED). A medical diagnosis code for COPD diagnosis was required to be confirmed by at least one of the following additional variables: a record of spirometry measurement, a hospitalisation with a discharge diagnosis for COPD or exacerbation of COPD (International Classification of Disease (ICD)-10 J44) or GP-reported Global Initiative For Chronic Obstructive Lung Disease (GOLD) classification recorded in 3 years prior, or 3 years following the CED. Patients were included in the study population if they had at least 12 months of data available before CED. Patients with alpha-1 antitrypsin deficiency (ICD-10 E88.0) were excluded.

### Exposure

A moderate exacerbation event was defined as an outpatient visit to the GP for COPD in combination with a dispensation of an oral corticosteroid within the 5 days following the visit and for a duration of ≥ 5 days and ≤ 15 days. The date of the GP visit was used as the first date of exposure (Day 1) to a moderate exacerbation.

A severe exacerbation was defined as a hospitalisation with a primary discharge diagnosis code of COPD or exacerbation of COPD (ICD-10 J44.0 or J44.1), or a secondary discharge diagnosis code for exacerbation of COPD (ICD-10 J44.1). The start date of exposure to a severe exacerbation was the date of hospital admission.

**Fig. 1:**
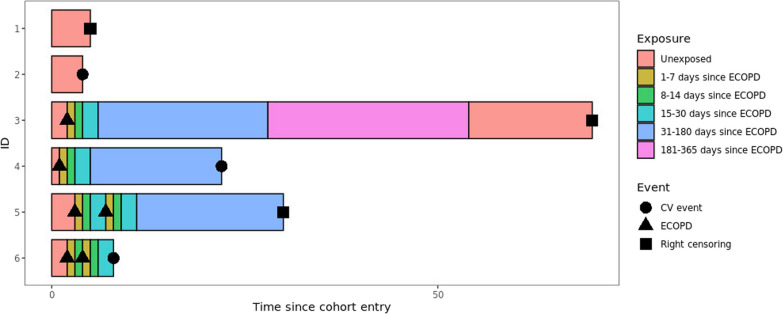
Cohort design of the present study. ECOPD: Exacerbation of chronic obstructive pulmonary disease; CV: cardiovascular; ID: patient identifier

Exposed time was set as the 365-day period starting on day 1 of an exacerbation event. Exposed time was divided into sub-periods of exposure, in which the hazard of the outcome could be assumed constant over time [8–10]. The following exposed time periods were used: 1–7 days, 8–14 days, 15–30 days, 31–180 days and 181–365 days following an exacerbation (Fig. 1). Upon the occurrence of a subsequent exacerbation, the exposed time periods started again at Day 1. As such, a patient could contribute data on a certain exposed time period more than once. Patients were considered unexposed during the time period prior to the first exacerbation and the time period post 365 days following an exacerbation. Patients who did not experience an exacerbation during follow-up were unexposed throughout the entire follow-up (Fig. 1).

### Follow-up

From the CED, each patient was followed until the earliest of (1) a first outcome of interest occurring after CED or (2) the end of data availability, or (3) end of study period on December 31st, 2019.

### Outcomes

Outcomes of interest included all-cause death and four categories of non-fatal severe CV events, defined as events that required a hospital admission with at least one overnight stay, with a primary or secondary discharge code (ICD-10; all coded provided on Additional file 1: Table S1) for (1) acute coronary syndrome (acute myocardial infarction and unstable angina, ACS), (2) heart failure (HF) decompensation, (3) cerebral ischaemia (including transient ischaemic attack (TIA)), or (4) arrhythmias (atrial fibrillation (AF) or other arrhythmias). To ascertain HF decompensation, only primary discharge codes were used reflecting the acuteness and decompensation of a chronic disease. For AF, events among patients with a history of AF were not considered since hospitalisations may occur in a non-urgent context for the management of AF. The mid-point of the hospitalisation period was considered as the date of the CV event, since the CV event may have occurred during the admission and not on the admission date. All-cause death was derived from death information from records in the PHARMO Data Network, supplemented with all-cause death data from the Dutch death registry. Several endpoints were defined: (a) time to the first outcome of interest (composite, i.e., a non-fatal severe event or all-cause death); (b) time to the first non-fatal severe CV event (to examine contribution of all-cause death to the composite risk); (c) time to the first event of each non-fatal CV event and all-cause death.

### Potential confounders

Baseline and time-dependent covariates were pre-specified as potential confounders. Baseline covariates that were defined over 12-month lookback period preceding CED were age, sex, socio-economic status (low/middle/high/unknown), and obesity (ICPC T82, ICD-10 E66, or body mass index > 30 kg/m^2^). Baseline covariates that were defined based on the entire available history (hospital ICD-9 or ICD-10 diagnosis or GP-reported ICPC diagnoses) included diabetes mellitus type 2, disorders of lipoprotein metabolism and other lipidaemias, ischaemic heart diseases, hypertensive diseases, heart failure, pulmonary oedema, pulmonary hypertension, venous thromboembolism, cerebrovascular disease, arrhythmia, asthma, chronic kidney disease or renal failure, mental illness and/or anxiety disorder. Time-dependent covariates were updated annually from CED onward unless otherwise stated and included COPD-related variables (number of GP visits in the last 12 months, number of prior moderate or severe exacerbations, and comedication use in the last 12 months (at least one prescription for long-acting inhaled bronchodilators, inhaled corticosteroids and their combination, short-acting inhalers, roflumilast and/or slow-release theophylline, any cardiac medication, any metabolic medication (ATC code listed in Additional file 1: Table S2). Smoking wasn’t included due to a substantial amount (> 50% of patients) of missing data.

### Statistical analyses

Baseline characteristics of the study population were described overall, and separately in patients who have, or have not exacerbated during study follow-up. Crude incidence rates (IR) of each outcome (composite, non-fatal CV events only, and individual categories) per 100 person-years were obtained, together with the corresponding 95% exact Poisson confidence intervals (CI).

Time-dependent Cox proportional hazards models were used with binary indicators of each exposed time period as time-dependent covariates, and the unexposed period as the reference. Separate models were fitted for (1) the association between exposure time periods following a moderate or severe exacerbation and each endpoint of interest, (2) for the separate association with moderate and severe exacerbations, and (3) for the association with a first, second and third exacerbation. All models were fitted with and without adjustment for all pre-specified fixed and time-varying confounders. For the analyses that examined the first, second and third exacerbation, exacerbations after the third were not evaluated. Analyses for the time to first severe CV event of each specific category, or all-cause death, were adjusted for the other “competing” CV events of interest, as time-varying confounders. All analyses of time to a first severe CV event were censored when patients died without experiencing the outcome of interest.

All data were analysed within Statistical Analysis Software (SAS) Enterprise Guide version 8.2 (SAS Institute Inc., Cary, NC, USA) and Windows using SAS version 9.4. Missing data were not imputed.

## Results

The source data contained a population of 1,636,513 individuals, including 8020 (0.5%) newly-diagnosed COPD patients (Additional file 1: Fig. S1). Mean age at COPD diagnosis was 65.4 years (SD, 10.7); 47% were female. Baseline characteristics of the study population are further presented in Table 1.

**Table 1:** Baseline characteristics of the cohort of newly diagnosed COPD patients identified between 2014 and 2018 in the PHARMO Data Network

Characteristic*	Overall	No exacerbation during follow-up	≥ 1 exacerbation during follow-up
	N = 8020	N = 5786	N = 2234
	n (%)	n (%)	n (%)
Age at CED (years), mean ± SD	65.4 ± 10.7	65.5 ± 10.6	67.6 ± 10.6
Female sex	3797 (47)	2681 (46)	1116 (50)
SES**			
Low	3255 (41)	2333 (40)	922 (41)
Middle	3124 (39)	2256 (39)	868 (39)
High	1635 (20)	1191 (21)	444 (20)
Unknown	6 (< 0.5)	6 (< 0.5)	0 (0)
Obesity**	919 (11)	684 (12)	235 (11)
Diabetes mellitus type-2	1076 (13)	715 (12)	361 (16)
Disorders of lipoprotein metabolism and other lipidaemias	1503 (19)	1113 (19)	390 (17)
Ischaemic heart disease	1393 (17)	959 (17)	434 (19)
Hypertensive disease	3001 (37)	2073 (36)	928 (42)
Heart failure	547 (7)	328 (6)	219 (10)
Pulmonary oedema	40 (< 0.5)	22 (< 0.5)	18 (1)
Pulmonary hypertension	32 (< 0.5)	17 (< 0.5)	15 (1)
Venous thromboembolism	310 (4)	210 (4)	100 (4)
Arrhythmia	864 (11)	563 (10)	301 (13)
Cerebrovascular disease	763 (10)	532 (9)	231 (10)
Asthma	940 (12)	676 (12)	264 (12)
Chronic kidney disease or renal failure	387 (5)	250 (4)	137 (6)
Severe mental illness and/or anxiety disorder	1564 (20)	1106 (19)	458 (21)
Number of GP visits**			
Mean ± SD	5.7 ± 7.0	5.4 ± 6.5	6.5 ± 7.9
Median (IQR)	4 (0–8)	4 (0–8)	4 (0–10)
Cardiac medication use**	3778 (47)	2423 (42)	1355 (61)
Metabolic medication use**	2722 (34)	1784 (31)	938 (42)

CED: Cohort entry date; COPD: chronic obstructive pulmonary disease; GP: General practitioner; IQR: Interquartile range; SD: Standard deviation; SES: Socio-economic status

^*^Based on entire available history, unless indicated otherwise; ** In 12-month lookback period prior to CED

During a median follow-up duration of 36 months (interquartile range 21–52 months), 2234 (28%) patients experienced at least one exacerbation; 1869 (23%) patients had at least one moderate exacerbation, and 743 (9%) patients had at least one severe exacerbation. Patients who were exposed to at least one exacerbation during study follow-up were more likely to be prescribed with COPD, cardiac and/or metabolic medication in the year preceding the first diagnosis of COPD (Table 1).

5270 (66%) patients were followed until 31 December 2019, whereas others were censored due to the end of data availability (n = 1658, 21%), a first non-fatal severe CV event (n = 631, 8%), and death without a prior severe CV event (n = 461, 6%). Among the 1092 patients who experienced an outcome of interest, the majority (n = 646, 59%) experienced the outcome prior to a first exacerbation or censoring; 33% (n = 360) had the CV event within the 1–365 days following an exacerbation, and 8% (n = 86) > 365 days following an exacerbation.

The frequency and proportion of patients who experienced at least 1 event of each outcome category separately (i.e., ignoring any CV event from other categories) are reported in Additional file 1: Table S2. ACS, HF decompensation, cerebral ischaemia and arrhythmias were each observed in about 2–3% of patients throughout follow-up, with HF decompensation being most common. Acute pulmonary oedema and cardiac arrest were rare (< 0.5%). In total, 567 patients (7%) died during follow-up, with or without a previous CV-event.

Crude incidence rates for the composite outcome are presented in Additional file 1: Table S3. Compared to unexposed periods, exposed periods following a moderate or severe exacerbation were associated with increased risk of the composite outcome. The increased risk decreased gradually over time, but persisted for the entire year [adjusted HR were 15.3 (95% CI 11.8–20.0) in days 1–7; 7.0 (95% CI 4.9–10.0) in days 8–14, 4.8 (95% CI 3.5–6.5) in days 15–30; 1.9 (95% CI 1.5–2.3) in days 31–180, and 1.3 (95% CI 1.0–1.8) in days 81–365] (Table 2).

**Table 2:** Hazard ratios for time to the composite outcome (non-fatal severe CV event or all-cause death), comparing exposed periods in the 365 days following the onset of an first exacerbation of COPD (moderate or severe/ moderate/ severe) to the non-exposure period

Time period	No. of first CV events of any type	Person years of follow-up	Unadjusted HR (95% CI)	Adjusted* HR (95% CI)
While patient is *unexposed*				
Prior to the first exacerbation or > 365 days since exposure	732	21,764	1	1
After the onset of an exacerbation (*moderate or severe*)				
1–7 days	82	98	24.1 (19.0–30.5)	15.3 (11.8–20.0)
8–14 days	38	96	11.0 (7.9–15.4)	7.0 (4.9–10.0)
15–30 days	51	204	7.4 (5.5–9.9)	4.8 (3.5–6.5)
31–180 days	124	1376	2.7 (2.2–3.3)	1.9 (1.5–2.3)
181–365 days	65	1069	1.8 (1.4–2.4)	1.3 (1.0–1.8)
After the onset of a *moderate* exacerbation				
1–7 days	10	76	3.7 (2.0–6.9)	2.5 (1.3–4.8)
8–14 days	11	75	4.0 (2.2–7.3)	2.7 (1.5–5.0)
15–30 days	21	161	3.9 (2.5–6.0)	2.6 (1.7–4.2)
31–180 days	80	1099	2.2 (1.7–2.8)	1.6 (1.3–2.1)
181–365 days	46	859	1.6 (1.2–2.2)	1.3 (0.9–1.7)
After the onset of a *severe* exacerbation				
1–7 days	72	22	91.5 (71.3–117.3)	48.6 (36.9–64.0)
8–14 days	27	21	37.2 (25.2–54.8)	20.0 (13.3–30.1)
15–30 days	30	43	20.1 (13.9–29.1)	11.3 (7.7–16.5)
31–180 days	44	275	4.8 (3.6–6.6)	2.8 (2.0–3.8)
181–365 days	19	209	2.8 (1.8–4.4)	1.6 (1.0–2.6)

CI: Confidence interval; CV: Cardiovascular; HR: Hazard ratio

^*^Adjusted for baseline covariates: year of cohort entry, age, sex, socio-economic status, obesity, type 2 diabetes, disorders of lipoprotein metabolism and other lipidaemias, ischaemic heart disease, hypertensive disease, heart failure, venous thromboembolism, cerebrovascular disease, arrhythmia, asthma, chronic kidney disease or renal failure, severe mental illness and/or anxiety disorder, and time-dependent covariates: number of GP visits in the last 12 months, number of prior moderate or severe exacerbations, long-acting and short-acting inhaled COPD drug use, roflumilast and/or theophylline, cardiac drugs and metabolic drugs

When examining the association for the time to the first non-fatal severe CV event, exposed periods following a moderate or severe exacerbation event were associated with increased risk of a non-fatal severe CV event, which persisted for the entire year (Table 3).

**Table 3:** Hazard ratios for time to the first non-fatal severe CV event together, or individually, or all-cause death, comparing exposed periods in the 365 days following a moderate or severe exacerbation of COPD to the non-exposure period

Time period	No. of first CV events of any type	Person years of follow-up	Unadjusted HR (95% CI)	Adjusted* HR (95% CI)
Any non-fatal severe CV event				
Unexposed period	593	21,803	1	1
1–7 days	54	98	25.9 (19.3–34.8)	20.7 (14.7–29.2)
8–14 days	23	96	10.7 (6.9–16.5)	8.6 (5.4–13.7)
15–30 days	23	204	5.2 (3.4–8.0)	4.3 (2.7–6.8)
31–180 days	65	1376	2.2 (1.7–2.9)	1.8 (1.3–2.5)
181–365 days	39	1069	1.7 (1.2–2.4)	1.5 (1.0–2.1)
ACS				
Unexposed period	132	21,838	1	1
1–7 days	12	99	15.2 (8.4–27.6)	9.9 (5.0–19.4)
8–14 days	6	97	8.7 (3.8–20.2)	7.7 (3.1–19.2)
15–30 days	8	208	6.1 (3.0–12.6)	4.8 (2.1–10.6)
31–180 days	19	1394	2.3 (1.4–3.7)	1.9 (1.1–3.2)
181–365 days	12	1084	1.7 (1.0–3.1)	1.5 (0.8–2.8)
HF decompensation				
Unexposed period	122	21,831	1	1
1–7 days	31	98	42.0 (28.1–62.9)	27.4 (17.3–43.4)
8–14 days	13	96	19.9 (11.0–36.2)	12.9 (6.8–24.5)
15–30 days	11	206	8.6 (4.6–16.1)	5.1 (2.7–9.9)
31–180 days	27	1385	3.5 (2.3–5.3)	2.3 (1.4–3.6)
181–365 days	12	1078	2.1 (1.2–3.8)	1.4 (0.8–2.6)
Cerebral ischaemia				
Unexposed period	126	21,854	1	1
1–7 days	4	99	4.6 (1.7–12.6)	3.5 (1.2–10.6)
8–14 days	1	98	1.5 (0.2–10.6)	1.1 (0.2–8.7)
15–30 days	4	209	3.0 (1.1–8.2)	2.4 (0.8–7.2)
31–180 days	7	1399	0.9 (0.4–1.8)	0.7 (0.3–1.7)
181–365 days	11	1087	1.8 (1.0–3.4)	1.5 (0.7–3.0)
Arrhythmia				
Unexposed period	128	21.855	1	1
1–7 days	10	99	9.7 (5.0–18.9)	9.4 (4.4–20.2)
8–14 days	6	97	9.0 (3.9–20.6)	8.8 (3.5–22.4)
15–30 days	5	208	3.7 (1.5–9.2)	4.0 (1.5–10.6)
31–180 days	11	1396	1.4 (0.7–2.5)	1.4 (0.7–2.9)
181–365 days	8	1085	1.3 (0.6–2.6)	1.3 (0.6–2.8)
All-cause death				
Unexposed period	346	21,878	1	1
1–7 days	37	99	14.1 (10.0–20.0)	5.4 (3.6–7.9)
8–14 days	18	98	10.2 (6.3–16.5)	4.3 (2.6–7.1)
15–30 days	39	209	11.3 (8.1–15.8)	5.3 (3.7–7.6)
31–180 days	89	1401	3.9 (3.1–5.0)	2.2 (1.7–2.9)
181–365 days	38	1090	2.2 (1.6–3.1)	1.3 (0.9–1.9)

ACS: Acute coronary syndrome; CI: Confidence interval; CV: Cardiovascular; HF: Heart failure; HR: Hazard ratio

^*^Adjusted for baseline covariates: year of cohort entry, age, sex, socio-economic status, obesity, type 2 diabetes, disorders of lipoprotein metabolism and other lipidaemias, ischaemic heart disease, hypertensive disease, heart failure, venous thromboembolism, cerebrovascular disease, arrhythmia, asthma, chronic kidney disease or renal failure, severe mental illness and/or anxiety disorder, and time-dependent covariates: number of GP visits in the last 12 months, number of moderate or severe prior exacerbations, long-acting and short-acting inhaled COPD drug use, roflumilast and/or theophylline, cardiac drugs and metabolic drugs, and a prior “competing” CV event

When analysing categories of severe CV events as separately, the risk of ACS, HF decompensation, and arrhythmia increased substantially following moderate or severe exacerbations. This risk decreased over time but remained significantly higher than in the unexposed period over the first 180 days. Adjusted HR were observed from 9.9 (95% CI 5.0–19.4) in days 1–7 to 1.9 (95% CI 1.1–1.3) in days 31–180 for acute coronary syndrome; adjusted HR from 27.4 (95% CI 17.3–43.4) in days 1–7 to 2.3 (95% CI 1.4–3.6) in days 31–180 for HF decompensation; and adjusted HR from 9.4 (95% CI 4.4–20.2) in days 1–7 to 4.0 (95% CI 1.5–10.6) in days 15–30 for arrhythmia. For cerebral ischaemia, the risk following an exacerbation was increased, on average, in the first 30 days following exacerbations, [adjusted HR 3.5 (95% CI 1.2–10.6) in days 1–7 to adjusted HR 2.4 (95% CI 0.8–7.2) in days 15–30], but less pronounced and estimated with uncertainty due to low event counts. The risk of all-cause death remained increased over the first 180 days after an exacerbation (Table 3).

After a moderate exacerbation, the risk of the composite outcome was increased and was stable over the first 30 days [adjusted HR 2.5 (95% CI 1.3–4.8) in days 1–7 to HR 2.6 (95% CI 1.7–4.2) in days 15–30]. After this 30-day period, the adjusted HR gradually decreased to 1.6 (95% CI 1.3–2.1) in the 31–180 days period and 1.3 (95% CI 0.9–1.7) in the 181–365 days period after a moderate exacerbation (Table 2).

After a severe exacerbation, the risk of the composite outcome increased substantially and remained significantly higher than in the overall unexposed periods up to the entire year [adjusted HR were 48.6 (95% CI 36.9–64.0) in days 1–7; 20.0 (95% CI 13.3–30.1) in days 8–14; 11.3 (95% CI 7.7–16.5) in days 15–30; 2.8 (95% CI 2.0–3.8) in days 31–180; and 1.6 (95% CI 1.0–2.6) in days 181–365].

Adjusted HR for day 1–7 period were 15.2 (95% CI 10.3–22.4) following first exacerbation, 22.3 (95% CI 14.3–34.6) following second exacerbation, and 20.2 (95% CI 10.7–38.0) following a third exacerbation (Table 4). Irrespective of whether the exacerbation was a first, second or third, the risk of the outcome decreased following exacerbation but remained significantly higher than the overall unexposed period over at least 180 days.

**Table 4:** Hazard ratios for time to the composite outcome (non-fatal severe CV event or all-cause death), comparing exposed periods in the 365 days following a first, second, and third exacerbation of COPD to the non-exposure period

Time period	No. of first CV events of any type	Person years of follow-up	Unadjusted HR (95% CI)	Adjusted* HR (95% CI)
After the onset of the *first* exacerbation (moderate or severe)				
1–7 days	33	43	20.0 (13.7–29.4)	15.2 (10.3–22.4)
8–14 days	20	42	12.1 (7.5–19.5)	9.3 (5.8–15.1)
15–30 days	16	91	4.9 (3.0–8.2)	3.9 (2.3–6.4)
31–180 days	49	704	2.0 (1.5–2.7)	1.6 (1.2–2.2)
181–365 days	38	640	1.8 (1.3–2.5)	1.5 (1.1–2.1)
After the onset of the *second* exacerbation (moderate or severe)				
1–7 days	21	20	30.9 (20.0–47.7)	22.3 (14.3–34.6)
8–14 days	6	20	8.9 (4.0–20.0)	6.5 (2.9–14.6)
15–30 days	9	43	6.2 (3.2–12.0)	4.6 (2.4–8.9)
31–180 days	31	289	3.3 (2.3–4.7)	2.6 (1.8–3.7)
181–365 days	12	226	1.6 (0.9–2.9)	1.3 (0.7–2.3)
After the onset of the *third* exacerbation (moderate or severe)				
1–7 days	10	11	28.2 (15.1–52.7)	20.2 (10.7–38.0)
8–14 days	3	11	8.5 (2.7–26.4)	5.8 (1.9–18.0)
15–30 days	7	23	9.3 (4.4–19.6)	6.6 (3.1–13.9)
31–180 days	15	148	3.1 (1.9–5.2)	2.3 (1.4–3.9)
181–365 days	7	96	2.3 (1.1–4.8)	1.6 (0.8–3.5)

CI: Confidence interval; CV: Cardiovascular; HR: Hazard ratio

^*^Adjusted for baseline covariates: year of cohort entry, age, sex, socio-economic status, obesity, type 2 diabetes, disorders of lipoprotein metabolism and other lipidaemias, ischaemic heart disease, hypertensive disease, heart failure, venous thromboembolism, cerebrovascular disease, arrhythmia, asthma, chronic kidney disease or renal failure, severe mental illness and/or anxiety disorder, and time-dependent covariates: number of GP visits in the last 12 months, number of prior moderate or severe exacerbations, long-acting and short-acting inhaled COPD drug use, roflumilast and/or theophylline, cardiac drugs and metabolic drugs

## Discussion

To our knowledge, this study is the first to report on the association between exacerbations of COPD and the risk of severe CV events or death in patients newly-diagnosed with COPD. We showed a substantially increased risk of a non-fatal severe CV event or death in the months following a moderate or severe exacerbation even in this cohort of patients with a new incident COPD diagnosis. This increase in risk was not constant over time: it was 15 times higher (for the composite outcome of a severe CV event or death) for 1–7 days following the onset of an exacerbation, and then decreased gradually during the subsequent periods of time, although risk remained significantly increased up to one year. The risk for individual types of non-fatal severe CV events was also increased following an exacerbation, including ACS, HF decompensation, cerebral ischaemia, and arrhythmias. The most prominent increase in risk was observed for HF decompensation.

Unique to this study is the fact that we specially explored CV risk after moderate exacerbations. When stratifying by the type of exacerbation, severe exacerbations were associated with a substantially increased risk of any severe CV event or death. The association for moderate exacerbations was less strong, though still significant for the first 180 days. Although the CV risk associated with moderate exacerbation was lower in magnitude compared to a severe exacerbation, moderate exacerbations are much more frequent, highlighting the clinical importance of this finding. Moreover, the elevated risk of a CV event or all-cause death was already present following the first exacerbation following new COPD diagnosis, again illustrating that COPD should be approached with urgency.

Our results confirm previous studies showing the risk of a severe CV event is increased in the days and months following an exacerbation [4, 11–13]. Previous studies were conducted in selected patients included in clinical trials, or used a self-controlled design selecting patients on the occurrence of a CV outcome [9, 14]. In this study with a narrow definition of exacerbation, over a quarter of COPD patients experienced an exacerbation within 3 years after diagnosis, exposing them to increased risk of CV events early in their disease trajectory. Our results thus highlight the burden of exacerbations at a very early stage of the disease. In addition, supporting evidence suggested that CV risk was higher following severe exacerbations than following moderate exacerbations [10, 12, 14, 15]. Importantly, we report here that the risk of severe CV events or all-cause death is immediately increased following a moderate exacerbation. The clinical relevance of experiencing significant risk of CV events following a first, moderate exacerbation is of paramount urgency, especially given that the majority of exacerbations in newly-diagnosed people living with COPD are of moderate severity. The importance of a single exacerbation for future cardiopulmonary risk has also recently been highlighted in the study by VanFleteren et al. showing that one exacerbation increased future risk for exacerbations and hospitalisation [16]. The fact that we showed that this already occurs early in the COPD disease trajectories leads to the hypothesis that early preventive treatment may be beneficial and this would be interesting for future research.

Information on specific cardiovascular events has been scarce up till now. Pooled results from a recent meta-analysis showed that the risk of stroke was 1.7 times higher and the risk of acute myocardial infarction was 2.4 times higher in the 1–3 months following an exacerbation [4]. Information on other CV events, such as HF decompensation and new-onset AF following exacerbation of COPD, has been limited. One study, based on a self-controlled case series reported an increased 30-days and 1-year incidences of AF and HF after 30 days and after 1 year following an exacerbation [11]. The current study design strengthens these findings in respect to granularity of the differential effects of exacerbations on the various CV events. We showed that people with COPD are most vulnerable in the first 30 days after an exacerbation throughout the whole spectrum of cardiovascular diseases. Risks for HF and ACS were highest suggesting that the acute effects of an exacerbation on the CV system could be drivers of such CV events. Our analyses adjusted for key observed confounders of the association between post-exacerbation time periods and the occurrence of a CV event, including comorbidities, among other factors. Nonetheless, unmeasured confounding may play a role and so our results should be interpreted as associations rather than causal effects.

By using real world GP data, our results increase the external validity of the findings of CV risk after exacerbations found in the randomized controlled trials. All citizens in the Netherlands are registered at a GP practice, except those living in a nursing home or hospice. The primary care information of patients included in the GP data can be considered representative for the general population [17], although linkage to hospital and pharmacy information could only be made for a subpopulation. Some limitations should be mentioned as well. First, some CV events could have been misclassified as exacerbation of COPD. For instance, a patient can be admitted for a severe exacerbation but actually have an HF decompensation, since symptoms of HF decompensation can mimic those of exacerbation of COPD. Such a misclassification, should it occurred, would have mainly impacted the day 1–7 period. Second, moderate exacerbations were strictly defined using the combination of a GP visit for COPD and a dispensation of oral corticosteroid. This strict definition tends to underrepresent moderate exacerbations: exacerbations managed with antibiotics only or modification of inhaled medications were missed by definition. Third, smoking history was missing for approximately 50% of the study population, and could therefore not be included as a potential confounder. Fourth, as cause of death is unknown in the PHARMO Data Network, it was not possible to include CV death in the composite outcome of severe CV events and all-cause death was included instead. Fifth, some less severe COPD patients might have been excluded from our study population because the diagnosis of COPD could not be confirmed.

This study has several clinical implications. The findings highlight the impact of COPD beyond respiratory function and symptoms. Patients with COPD are at high risk of cardiopulmonary events such as exacerbations and CV events. It emphasizes the importance of a treatment paradigm that includes prevention, early monitoring, and treatment of this cardiopulmonary risk from the start of COPD diagnosis. In clinical practice, severe CV events are often under-diagnosed in patients experiencing exacerbations of COPD [18–20]. There is a need to optimize COPD management across specialties focusing on reducing risk of exacerbations. Future research should further clarify whether the number of exacerbations affect the magnitude of the CV risk, and should further examine the risk of cerebral ischaemia following exacerbations of COPD.

## Conclusions

In this population of newly diagnosed COPD patients, we observed a substantial increase in the risk of severe CV events or all-cause death after a moderate or severe exacerbation. The increase in risk was strongest in the month after the onset of an exacerbation and concerned all categories of severe CV events, i.e., ACS, HF, arrhythmias, or cerebral ischemia. These findings highlight the high cardiovascular risk in people living with COPD, even among those that are early in their disease trajectory.

### Supplementary Information

**Additional file 1: Figure S1.** Flowchart of patient selection. **Table S1.** Codes used for identification of outcomes, and covariate. **Table S2.** Outcomes during follow-up in the study population of newly diagnosed COPD patients. **Table S3.** Crude incidence rates for the first outcome of interest (non-fatal severe CV events or all-cause death) in exposed periods in the 365 days following an exacerbation of COPD (moderate or severe, moderate, severe, first, second, and third) and in the unexposed period.

## Data Availability

The datasets generated and/or analysed during the current study are not publicly available but are available from the corresponding author on reasonable request and after approval of the Compliance Committee of Stichting Informatievoorziening voor Zorg en ONderzoek.

## Abbreviations

ACS: Acute coronary syndrome
AF: Atrial fibrillation
CED: Cohort entry date
CI: Confidence interval
COPD: Chronic obstructive pulmonary disease
CV: Cardiovascular
EXACOS-CV: EXAcerbations of COPD and their OutcomS on CardioVascular disease
GOLD: Global initiative for Chronic Obstructive Lung Disease
GP: General practitioner
HF: Heart failure decompensation
HR: Hazard ratio
ICD: International Classification of Disease
ICPC: International Classification of Primary Care
TIA: Transient ischaemic attack
