# Supplementary material for: Risk of cardiovascular events after an exacerbation of chronic obstructive pulmonary disease: results from the EXACOS-CV cohort study using the PHARMO Data Network in the Netherlands
Source: Respir Res. 2023 Nov 21;24:293. doi: 10.1186/s12931-023-02601-4 (PMC10662240; doi:10.1186/s12931-023-02601-4)
Supplement: Supplementary file 1 — Additional file 1: Figure S1. Flowchart of patient selection. Table S1. Codes used for identification of outcomes, and covariate. Table S2. Outcomes during follow-up in the study population of newly diagnosed COPD patients. Table S3. Crude incidence rates for the first outcome of interest (non-fatal severe CV events or all-cause death) in exposed periods in the 365 days following an exacerbation of COPD (moderate or severe, moderate, severe, first, second, and third) and in the unexposed period. [file 12931_2023_2601_MOESM1_ESM.docx]

| **Source population**  All patients in the general practitioner data, hospital data, and the out-patient pharmacy data  between January 1, 2014 and December 31, 2018  N = 1,636,513 | | | | | | |
| --- | --- | --- | --- | --- | --- | --- |
|  |  |  |  |  |  |  |
|  |  |  | | | | No incident diagnosis of COPD  N = 1,623,540 (99%) |
|  |  |  | | | |  |
|  |  |  |  |  |  |  |
| Patients with an incident diagnosis of COPD  N = 12,973 | | | | |  |  |
|  |  |  |  |  |  |  |
|  |  |  |  |  |  |  |
|  |  |  |  |  |  | No GOLD classification, spirometry or COPD hospitalisation  N = 4,109 (32%) |
|  |  |  |  |  |  |  |
|  |  |  |  |  |  |  |
| Patients with an incident diagnosis of COPD  and  a GOLD classification, spirometry, or COPD hospitalisation*  N = 8,864 | | | | |  |  |
|  |  |  |  |  |  |  |
|  |  |  |  |  |  | Age <40 years at CED  N = 151 (2%) |
|  |  |  |  |  |  |  |
|  |  |  |  |  |  |  |
| Age ≥40 years at CED  N = 8,713 | | | | |  |  |
|  |  |  |  |  |  |  |
|  |  |  |  |  |  | <12 months of data  available before CED  N = 688 (8%) |
|  |  |  |  |  |  |  |
|  |  |  |  |  |  |  |
| ≥12 months of data available before CED  N = 8,025 | | | | |  |  |
|  |  |  |  |  |  |  |
|  |  |  |  |  |  | Excluded: patients with AAT-deficiency  N = 5 (<0.5%) |
|  |  |  |  |  |  |  |
|  |  |  |  |  |  |  |
| **Study population**  N = 8,020 | | | | | | |

**Figure S1:** Flowchart of patient selection

Abbreviations: AAT= Alpha-1 antitrypsin; CED= Cohort entry date; COPD= Chronic obstructive pulmonary disease; GOLD= Global initiative for chronic obstructive lung disease

* An incident diagnosis of COPD was defined as a diagnosis record of COPD and a GP-reported GOLD classification, a spirometry measurement, or a COPD hospitalisation in the 3 years prior or following CED

**Table S1:** Codes used for identification outcomes, and covariates

| **Coding System** | **Diagnosis** | **Code** | **Description** |
| --- | --- | --- | --- |
| **Outcomes of interest** | |  |  |
| ICD-10 | Acute myocardial infarction | I21 | Acute myocardial infarction |
|  | Acute myocardial infarction | I22 | Subsequent ST elevation (STEMI) and non-ST elevation (NSTEMI) myocardial infarction |
|  | Acute myocardial infarction | I25.6 | Silent myocardial ischemia |
|  |  |  |  |
|  | Unstable angina | I20.0 | Unstable angina pectoris |
|  | HF decompensation | I50 | Heart failure |
|  | HF decompensation | I11.0 | Hypertensive heart disease with heart failure |
|  | HF decompensation | I13.0 | Hypertensive heart and chronic kidney disease with heart failure and stage 1 through stage 4 chronic kidney disease, or unspecified chronic kidney disease |
|  | HF decompensation | I13.2 | Hypertensive heart and chronic kidney disease with heart failure and with stage 5 chronic kidney disease, or end stage renal disease |
|  | Pulmonary oedema | J81 | Pulmonary oedema |
|  | Atrial fibrillation | I48 | Atrial fibrillation or flutter |
|  | Other arrhythmias | I46 | Cardiac arrest |
|  | Other arrhythmias | I49 | Other cardiac arrhythmias |
|  | Other arrhythmias | I47 | Paroxysmal tachycardia |
|  | Cerebral ischaemia | I63 | Cerebral infarction, unspecified |
|  | Transient ischaemic attack | G45 | Transient cerebral ischaemic attack, unspecified |
| **Comorbidities** |  |  |  |
| ICD-9* | Diabetes mellitus | 250 | Diabetes mellitus |
|  | Disorders of lipoprotein metabolism and other lipidaemias | 272 | Disorders of lipoprotein metabolism and other lipidaemias |
|  | Ischaemic heart disease | 410 | Acute myocardial infarction |
|  | Ischaemic heart disease | 411 | Other acute and subacute forms of ischaemic heart disease |
|  | Ischaemic heart disease | 412 | Old myocardial infarction |
|  | Ischaemic heart disease | 413 | Angina pectoris |
|  | Ischaemic heart disease | 414 | Other forms of chronic ischaemic heart disease |
|  | Hypertensive disease | 401 | Essential hypertension |
|  | Hypertensive disease | 402 | Hypertensive heart disease |
|  | Hypertensive disease | 403 | Hypertensive renal disease |
|  | Hypertensive disease | 404 | Hypertensive heart and renal disease |
|  | Hypertensive disease | 405 | Secondary hypertension |
|  | Heart failure | 428 | Heart failure |
|  | Heart failure | 402.x1 | Hypertensive heart failure |
|  | Pulmonary oedema | 514 | Pulmonary oedema, chronic or unspecified |
|  | Pulmonary oedema | 518.4 | Pulmonary oedema, acute |
|  | Pulmonary oedema | 428.1 | Acute pulmonary oedema, with mention of heart disease or failure |
|  | Pulmonary hypertension | 416.0 | Primary pulmonary hypertension |
|  | Pulmonary hypertension | 416.8 | Other secondary pulmonary hypertension |
|  | Pulmonary hypertension | 416.9 | Chronic pulmonary heart disease, unspecified |
|  | Venous thromboembolism | 451 | Phlebitis and thrombophlebitis |
|  | Venous thromboembolism | 452 | Portal vein thrombosis |
|  | Venous thromboembolism | 453 | Other venous embolism and thrombosis |
|  | Venous thromboembolism | 415 | Pulmonary embolism |
|  | Venous thromboembolism | 437.6 | Nonpyogenic thrombosis of intracranial venous sinus |
|  | Venous thromboembolism | 639.6 | Embolism following ectopic and molar pregnancy |
|  | Venous thromboembolism | 6713 | Deep phlebothrombosis in pregnancy |
|  | Venous thromboembolism | 6714 | Deep phlebothrombosis in the puerperium |
|  | Venous thromboembolism | 6715 | Other phlebitis and thrombosis in pregnancy and the puerperium |
|  | Venous thromboembolism | 6732 | Obstetric thromboembolism |
|  | Cerebrovascular disease | 430-438 | Cerebrovascular diseases |
|  | Arrhythmia | 427 | Cardiac dysrhythmia |
|  | Asthma | 493 | Asthma |
|  | Chronic kidney disease | 585 | Chronic kidney disease |
|  | Chronic kidney disease | 403 | Hypertensive chronic kidney disease |
|  | Chronic kidney disease | 404 | Hypertensive heart and chronic kidney disease |
|  | Mental illness and/or anxiety disorders | 296 | Affective psychosis |
|  | Mental illness and/or anxiety disorders | 295 | Schizophrenic disorders |
|  | Mental illness and/or anxiety disorders | 300 | Neurotic disorders |
|  |  | 311 | Depression |
| ICD-10 | Obesity | E66 | Obesity |
|  | Diabetes mellitus | E10 | Insulin-dependent diabetes mellitus |
|  | Diabetes mellitus | E11 | Non-insulin-dependent diabetes mellitus |
|  | Diabetes mellitus | E12 | Malnutrition-related diabetes mellitus |
|  | Diabetes mellitus | E13 | Other specified diabetes mellitus |
|  | Diabetes mellitus | E14 | Unspecified diabetes mellitus |
|  | Disorders of lipoprotein metabolism and other lipidaemias | E78 | Disorders of lipoprotein metabolism and other lipidaemias |
|  | Ischaemic heart disease | I20 | Angina pectoris |
|  | Ischaemic heart disease | I21 | Acute myocardial infarction |
|  | Ischaemic heart disease | I22 | Subsequent myocardial infarction |
|  | Ischaemic heart disease | I23 | Certain current complications |
|  | Ischaemic heart disease | I24 | Other acute ischaemic heart diseases |
|  | Ischaemic heart disease | I25 | Chronic ischaemic heart disease |
|  | Hypertensive diseases | I10 | Essential (primary) hypertension |
|  | Hypertensive diseases | I11 | Hypertensive heart disease |
|  | Hypertensive diseases | I12 | Hypertensive renal disease |
|  | Hypertensive diseases | I13 | Hypertensive heart and renal disease |
|  | Hypertensive diseases | I15 | Secondary hypertension |
|  | HF | I50 | Heart failure |
|  | Pulmonary oedema | J81 | Pulmonary oedema |
|  | Pulmonary hypertension | I27.0 | Primary pulmonary hypertension |
|  | Pulmonary hypertension | I27.2 | Other secondary pulmonary hypertension |
|  | Venous thromboembolism | I80 | Phlebitis and thrombophlebitis |
|  | Venous thromboembolism | I81 | Portal vein thrombosis |
|  | Venous thromboembolism | I82 | Other venous embolism and thrombosis |
|  | Venous thromboembolism | I26 | Pulmonary embolism |
|  | Venous thromboembolism | O08.2 | Embolism following ectopic and molar pregnancy |
|  | Venous thromboembolism | O22.3 | Deep phlebothrombosis in pregnancy |
|  | Venous thromboembolism | O87.1 | Deep phlebothrombosis in the puerperium |
|  | Venous thromboembolism | O88.2 | Obstetric thromboembolism |
|  | Cerebrovascular disease | I60-I69 | Cerebrovascular diseases |
|  | Arrhythmia | I48 | Atrial fibrillation and flutter |
|  | Arrhythmia | I49 | Other cardiac arrythmias |
|  | Arrhythmia | I46 | Cardiac arrest |
|  | Arrhythmia | I47 | Paroxysmal tachycardia |
|  | Asthma | J45 | Asthma |
|  | Asthma | J46 | Status asthmaticus |
|  | Chronic kidney disease | N17-N19 | Acute kidney failure and chronic kidney disease |
|  | Chronic kidney disease | I12 | Hypertensive renal disease |
|  | Chronic kidney disease | I13.1 | Hypertensive heart and chronic kidney disease without heart failure |
|  | Chronic kidney disease | I13.2 | Hypertensive heart and chronic kidney disease with heart failure and with stage 5 chronic kidney disease, or end stage renal disease |
|  | Mental illness and/or anxiety disorders | F33 | Recurrent depressive disorder |
|  | Mental illness and/or anxiety disorders | F32 | Depressive episode |
|  | Mental illness and/or anxiety disorders | F34 | Persistent mood [affective] disorders |
|  | Mental illness and/or anxiety disorders | F31 | Bipolar affective disorder |
|  | Mental illness and/or anxiety disorders | F20 | Schizophrenia |
|  | Mental illness and/or anxiety disorder | F41 | Other anxiety disorders |
|  | Mental illness and/or anxiety disorders | F40 | Phobic anxiety disorders |
| ICPC-1 | Obesity | T82 | Obesity |
|  | Diabetes mellitus | T90 | Diabetes mellitus |
|  | Disorders of lipoprotein metabolism and other lipidaemias | T93 | Disorders of lipoprotein metabolism |
|  | Ischaemic heart disease | K74 | Angina pectoris |
|  | Ischaemic heart disease | K75 | Acute myocardial infaction |
|  | Ischaemic heart disease | K76 | Other/chronic ischaemic heart disease |
|  | Hypertensive diseases | K86 | Essential hypertension |
|  | Hypertensive disease | K87 | Secondary hypertension |
|  | Heart failure | K77 | Chronic decompensatio cordis |
|  | Pulmonary oedema | - | - |
|  | Pulmonary hypertension | K82 | Pulmonary hypertension |
|  | Venous thromboembolism | K94 | Thrombophlebitis /phlebothrombosis |
|  | Venous thromboembolism | K93 | Pulmonary embolism |
|  | Cerebrovascular disease | K90 | Cerebrovascular accident |
|  | Cerebrovascular disease | K89 | Transient ischaemic attack |
|  | Arrhythmia | K78 | Atrial fibrillation and flutter |
|  | Asthma | R96 | Asthma |
|  | Asthma | R96.01 | Hyperreactive airways |
|  | Asthma | R96.02 | Allergic asthma |
|  | Chronic kidney disease | U99.01 | Renal dysfunction |
|  | Mental illness and/or anxiety disorders | P76 | Depression |
|  | Mental illness and/or anxiety disorders | P73 | Affective psychosis |
|  | Mental illness and/or anxiety disorders | P72 | Schizofrenia |
|  | Mental illness and/or anxiety disorders | P74 | Anxiety disorder |
| WCIA | Smoking | 1739 | Smoking |
| **Medication** |  |  |  |
| ATC | ICS | R03BA | Glucocorticoids |
|  | ICS | R03AK06 | Salmeterol/ fluticasone |
|  | ICS | R03AK07 | Formoterol/ budesonide |
|  | ICS | R03AK08 | Formoterol/ beclometason |
|  | ICS | R03AK09 | Formoterol/ mometasone |
|  | ICS | R03AK10 | Vilanterol/ fluticasone |
|  | ICS | R03AK11 | Formoterol/ fluticason |
|  | ICS | R03AK12 | Budesonide / salmeterol |
|  | ICS | R03AK13 | Salbutamol/ beclometason |
|  | ICS | R03AK14 | Indacaterol/ mometason |
|  | ICS | R03AL08 | Vilanterol/ umeclidinium/ fluticasone |
|  | ICS | R03AL09 | Beclametason/ formoterol/ glycopyrronium |
|  | ICS | R03AL11 | Formoterol/ blycopyrronium/ budesonide |
|  | ICS | R03AL12 | Indacaterol/ glycopyrronium/ mometason |
|  | SABA | R03AC02 | Salbutamol |
|  | SABA | R03AC03 | Terbutaline |
|  | SABA | R03AL02 | Salbutamol/ ipratropium |
|  | SAMA | R03BB01 | Ipratropium |
|  | LABA | R03AK06 | Salmeterol/ fluticasone |
|  | LABA | R03AK07 | Formoterol/ budesonide |
|  | LABA | R03AK11 | Formoterol/ fluticasone |
|  | LABA | R03AC13 | Formoterol |
|  | LABA | R03AC12 | Salmeterol |
|  | LABA | R03AL01 | Fenoterol/ ipratropium |
|  | LABA | R03AK10 | Vilanterol/ fluticasone |
|  | LAMA | R03BB04 | Tiotropium |
|  | Roflumilast | R03DX07 | Roflumilast |
|  | Theophylline | R03DA04 | Theophylline |
|  | Cardiac drugs | B01 | Antithrombotic agents |
|  | Cardiac drugs | C01B | Antiarrhythmics, class I and II |
|  | Cardiac drugs | C01AA | Digitalis glycosides |
|  | Cardiac drugs | C02 | Antihypertensives |
|  | Cardiac drugs | C03 | Diuretics |
|  | Cardiac drugs | C07 | Beta blocking agents |
|  | Cardiac drugs | C08 | Calcium channel blockers |
|  | Cardiac drugs | C09 | Agents acting on the renin-angiotensin system |
|  | Metabolic drugs | C10 | Lipid modifying agents |
|  | Metabolic drugs | A10 | Antidiabetic agents |

Abbreviations: AAT= Alpha-1 antitrypsin; ATC= Anatomical therapeutic chemical; HF= Heart failure; ICD= International classification of disease; ICPC= International classification of primary care; ICS= Inhaled corticosteroids; LABA= Long-Acting Beta-2 agonists; LAMA= Long-acting muscarinic antagonistis; SABA= Short-acting bata-2 agonists; SAMA= Short-acting muscarinic antagonists

*ICD-9 was used only for covariates for which the entire history was needed

**Table S2:** Outcomes during follow-up in the study population of newly diagnosed COPD patients

| **Outcomes** | **Study population**  **N=8,020** |
| --- | --- |
|  | **n (%)** |
| Patients with any CV event of interest* | 1,092 (14) |
| Any ACS | 198 (2) |
| Acute myocardial infarction | 160 (2) |
| Unstable angina | 49 (1) |
| HF decompensation | 230 (3) |
| (Congestive) HF | 228 (3) |
| Acute pulmonary oedema | <5** (<0.5) |
| Cerebral ischaemia | 163 (2) |
| Ischaemic stroke | 106 (1) |
| Transient ischaemic attacks | 60 (1) |
| Arrhythmias | 177 (2) |
| Atrial fibrillation | 101 (1) |
| Other cardiac arrhythmias | 133 (2) |
| Cardiac arrest | 22 (<0.5) |
| Death (all-cause) | 567 (7) |

Abbreviations: ACS= Acute coronary syndrome; CV= Cardiovascular; HF= Heart failure

*The sum across different CV events is higher than the total number of first CV events of any type, since patients may have had more than one type of CV events. **To limit patient identifiability, the actual number of observations cannot be provided.

**Table S3:** Crude incidence rates for the first outcome of interest (non-fatal severe CV events or all-cause death) in exposed periods in the 365 days following an exacerbation of COPD (moderate or severe, moderate, severe, first, second, and third) and in the unexposed period

| **Time period** | **Crude incidence rate per 100 person years (95% CI)** |
| --- | --- |
| **While patient is unexposed** | |
| Prior to the first exacerbation or >365 days since exposure | 3.4 (3.1-3.6) |
| **After the onset of an exacerbation (moderate or severe)** | |
| 1-7 days | 84.1 (66.9-104.4) |
| 8-14 days | 39.7 (28.1-54.6) |
| 15-30 days | 25.0 (18.6-32.8) |
| 31-180 days | 9.0 (7.5-10.8) |
| 181-365 days | 6.1 (4.7-7.8) |
| **After the onset of a moderate exacerbation** | |
| 1-7 days | 13.2 (6.4-24.4) |
| 8-14 days | 14.7 (7.3-26.3) |
| 15-30 days | 13.0 (8.1-19.9) |
| 31-180 days | 7.3 (5.8-9.1) |
| 181-365 days | 5.4 (3.9-7.1) |
| **After the onset of a severe exacerbation** | |
| 1-7 days | 327.0 (255.9-411.9) |
| 8-14 days | 130.3 (85.9-189.5) |
| 15-30 days | 69.8 (47.1-99.6) |
| 31-180 days | 16.0 (11.6-21.5) |
| 181-365 days | 9.1 (5.5-14.2) |
| **After the onset of a first exacerbation (moderate or severe)** | |
| 1-7 days | 77.5 (53.4-108.9) |
| 8-14 days | 47.8 (29.2-73.9) |
| 15-30 days | 17.6 (10.0-28.6) |
| 31-180 days | 7.0 (5.2-9.2) |
| 181-365 days | 5.9 (4.2-8.1) |
| **After the onset of the second exacerbation (moderate or severe)** | |
| 1-7 days | 105.2 (65.1-160.9) |
| 8-14 days | 30.6 (11.2-66.6) |
| 15-30 days | 21.1 (9.6-40.0) |
| 31-180 days | 10.7 (7.3-15.2) |
| 181-365 days | 5.3 (2.7-9.3) |
| **After the onset of the third exacerbation (moderate or severe)** | |
| 1-7 days | 89.6 (43.0-164.8) |
| 8-14 days | 27.5 (5.7-80.2) |
| 15-30 days | 30.4 (12.2-62.6) |
| 31-180 days | 10.1 (5.7-16.7) |
| 181-365 days | 7.3 (2.9-15.0) |

Abbreviations: CI= Confidence interval
